# Effect of thermal treatment at high temperature on phase stability and transformation of Yb_2_O_3_ and Y_2_O_3_ co-doped ZrO_2_ ceramics

**DOI:** 10.1038/s41598-022-13705-0

**Published:** 2022-06-15

**Authors:** Zheng Cao, Shengli An, Xiwen Song

**Affiliations:** 1 School of Metallurgy and Ecological Engineering, University of Science and Technology Beijing, 30 Xueyuan Road, Beijing, 100083 China; 2 Inner Mongolia Key Laboratory of Advanced Ceramic Materialss and Devices, Inner Mongolia University of Science and Technology, 7 Arding Street, Baotou, 014010 China; 3 School of Materials and Metallurgy, Inner Mongolia University of Science and Technology, 7 Arding Street, Baotou, 014010 China

**Keywords:** Materials science, Aerospace engineering

## Abstract

Y_2_O_3_ doped ZrO_2_ (YSZ) ceramic material is used to protect alloy components worked in high-temperature. But its phase transformation between tetragonal phase and monoclinic phase occurred at 1150 °C leads to YSZ invalid. Therefore, enhancing the phase stability of YSZ is necessary for meeting the demands of the development of thermal barrier coatings (TBC). In this study, X-ray diffraction and Raman spectra were used to explore the phase stability and phase transformation of Yb_2_O_3_ and Y_2_O_3_ co-doped ZrO_2_ (YbYSZ) ceramics after heat treatment at 1300 °C with different times. The stability of tetragonal phase is improved by tetragonality decreasing with Yb^3+^ doped. Simultaneously, the incorporation of Yb^3+^ leads to O–O coupling, which is beneficial for increasing the concentration of oxygen vacancies near the substituted ions, thereby improving the stability of the crystal. The 6.5YbYSZ ceramic has the best stability after heat treatment at 1300 °C for different times.

## Introduction

More efficient engineering components for applications in the energy, automotive, aerospace, electronics, and power industries are desired in the current competitive world economy. Thermal barrier coatings (TBCs) are usually used to protect those components that operate in high-temperature, corrosion, or other harsh environments^1,2^. TBCs are composed of two important layers: metal bond coat and ceramic top coat. Metal bond coat always uses MCrAlY (M = Ni, Co, Ni + Co, etc.) alloy to protect the components from oxidation and corrosion, while ceramic top coat acts as an insulator^3,4^. Being in direct contact with the harsh working environment, ceramic top-coat should have lower thermal diffusivity, better performance on phase stability and thermal shock resistance during thermal cycling, as well as better oxidation and corrosion resistance^5,6^. 6–8 wt% Y_2_O_3_ partially stabilized ZrO_2_ (YSZ) as the most promising choice of ceramic top coat shows outstanding comprehensive performance in thermal conductivity, phase stability, and other aspects^5,7,8^. However, when the operating ambient temperature exceeds 1200 °C, the tetragonal (t) phase transforms to the monoclinic (m) phase, which is accompanied by a volume expansion of 3–5%, leading to detrimental cracks in coatings^9,10^. Moreover, at high temperatures (exceeds 1200 °C), the pores inside the YSZ coatings undergo shrinkage, particularly those perpendicular to the heat flow, thus resulting in a significant increase in the thermal conductivity of TBCs^11–14^.

Therefore, the research and development of lower thermal conductivity and more stable top coat ceramic materials at high temperatures are urgently needed for the development of new-generation gas turbines. Numerous studies have shown that doping rare earth oxides (RE_2_O_3_) with different atomic masses or radii into YSZ systems is an effective method for improving thermal insulation performance and high-temperature phase stability^15–19^. Stecura et al.^20^ explored the thermal cycle life of Yb_2_O_3_-stabilized ZrO_2_ system at 1120 °C and found that the thermal cycle failure modes of Yb_2_O_3_-ZrO_2_ and Y_2_O_3_-ZrO_2_ were similar, but the thermal cycle life of Yb_2_O_3_-ZrO_2_ was significantly better than that of YSZ. By comparing the phase stability of Yb_2_O_3_ and Y_2_O_3_ co-stabilized ZrO_2_ at 1450 °C, Caireny et al.^21^ found that the addition of Yb could efficiently improve the phase stability. Jing et al.^22^ studied 3–10 mol% Yb_2_O_3_-stabilized ZrO_2_ ceramics and found that the ceramics consisted with the metastable tetragonal phase (t′) and had lower thermal conductivity. Leilei et al.^23^ systematically studied the effects of Yb_2_O_3_ and Y_2_O_3_ co-doped in ZrO_2_ on the phase stability and thermal conductivity. Their results showed that co-doped ZrO_2_ had better phase stability and lower thermal conductivity than those of Yb_2_O_3_- or Y_2_O_3_-doped ZrO_2_ ceramics. Lei and his colleagues^16,24,25^ prepared 1 mol% RE_2_O_3_ (RE = La, Nd, Gd) and 1 mol% Yb_2_O_3_–co-doped YSZ (1RE1Yb–YSZ) ceramics and 3.5 mol% RESZ (RE = Dy, Y, Er, Yb) ceramics by a chemical co-precipitation method. They found that all the prepared ceramics were composed of t′ phase. The phase stability and thermal conductivity of 1RE1Yb–YSZ decreased with the increase of RE^3+^ ion radius, whereas the fracture toughness of 3.5 mol% RESZ showed the opposite trend. In addition, the corrosion resistance of a GdYb-YSZ ceramic was better than that of YSZ.

This study is based on the better performance of YbYSZ system. X-ray diffraction and Raman spectra are used to explore the phase composition and phase transformation of ceramic samples heat-treated at 1300 °C for different times.

## Experimental procedure

### Material preparation

*x* mol% YbO_1.5_ − (8.5-*x*) mol% YO_1.5_ − ZrO_2_ (*x* = 0, 2.5, 4.5, 6.5, and 8.5, denoted as *x*YbYSZ) ceramics were prepared by a solid-state reaction method. Y_2_O_3_, Yb_2_O_3_, and ZrO_2_ (99.9%, Zhongnuo New Material Technology Co. Ltd.) were used as raw materials. All oxide powders were calcined at 800 °C for 5 h to eliminate the influence of absorbed water before mixed. Then, the oxides weighed with stoichiometric ratios were milled by two steps. First step was to mix-up all raw materials and pulverize the oxides to micron scale by ball milling. The second step was to further refine the precursor mixed oxide slurry, which was milled in a high-energy ball mill at 2300 rpm, 2500 rpm, and 2700 rpm for 3 h respectively to obtain a nanoscale mixture. The slurry after two grinding steps was completely dried at 80 °C and then sintered at 1450 °C for 3 h to obtain the initial ceramic samples.

### Experiment of heat treatment

All initial ceramic samples were heat-treated in a muffle furnace at 1300 °C for 9, 33, 93, 143, 208, 287 and 358 h and then cooled to room temperature at a rate of 10°/min.

### Structural characterization and analysis

X-ray diffraction (XRD, Rigaku Smart Lab II, Japan) and Raman spectroscopy (Raman, Horiba, Japan) were used to identify the phase composition and structure of initial ceramic samples and heat-treated ceramic samples. The XRD scans from 20° to 80° at a scan rate of 5°/min with Cu Kα radiation (λ = 0.15418 nm). The Raman scans from 100/60 cm^−1^ to 800 cm^−1^ with a green laser (532 nm).

## Results and discussion

### Phase composition and structure of initial ceramic samples

**Figure 1:**
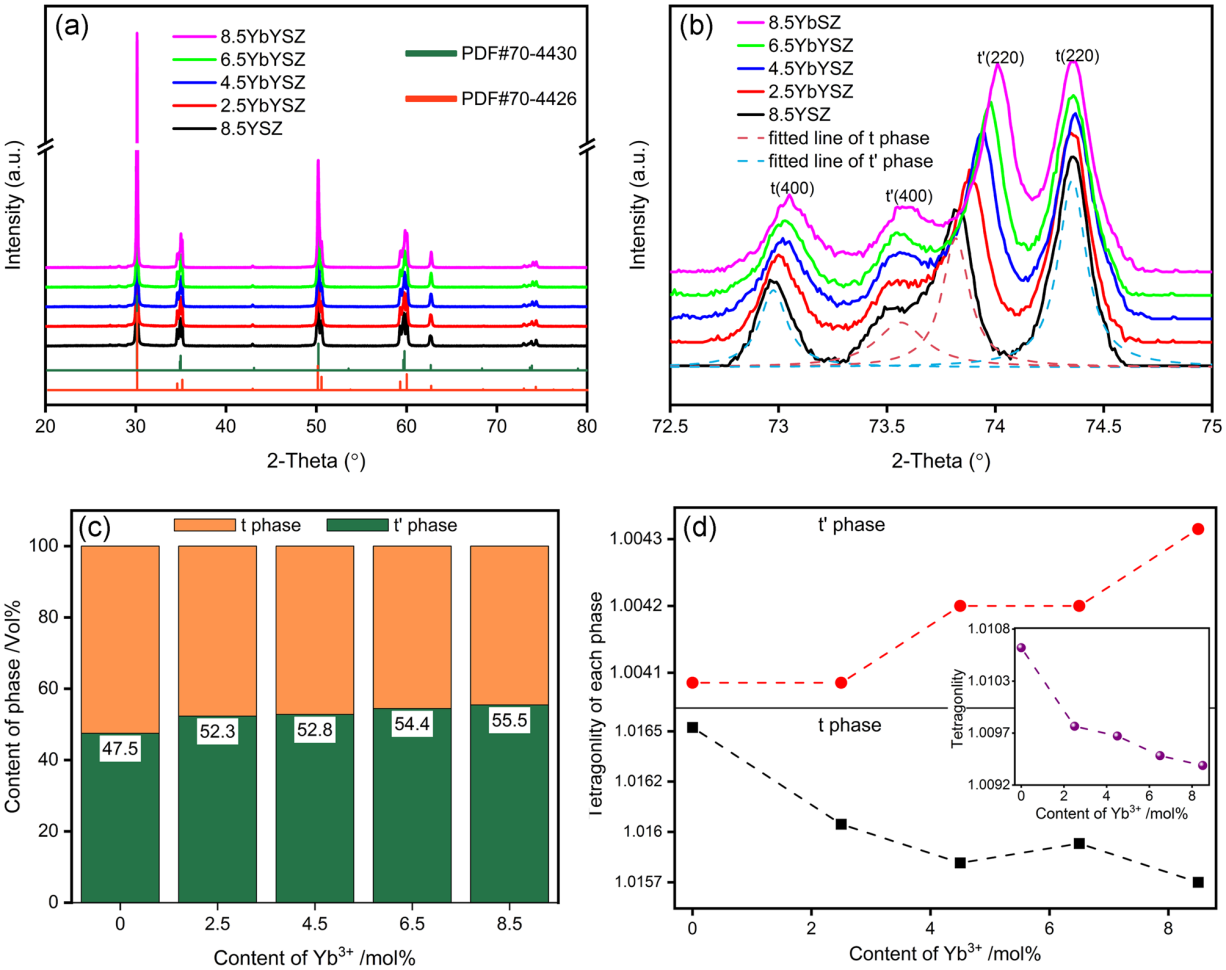
XRD patterns of *x*YbYSZ (*x* = 0, 2.5, 4.5, 6.5, and 8.5): (**a**) 2θ = 20°–80° at a scan rate of 5°/min, (**b**) 2θ = 72.5°–75° at a scan rate of 1°/min; (**c**) phase content and (**d**) tetragonality of t phase and t’ phase.

The XRD patterns of *x*YbYSZ (*x* = 0, 2.5, 4.5, 6.5, and 8.5) ceramics are presented in Fig. 1. As shown in Fig. 1a, the diffraction peaks correspond to two different tetragonal-related PDF cards (PDF Nos. 70-4426 and 70-4430). The PDF cards can be defined as metastable (PDF#4430, t′) and stable (PDF#4426, t) tetragonal zirconia phase due to the difference in lattice parameters^26,27^. Therefore, the XRD patterns suggest that Yb^3+^ and Y^3+^ have completely dissolved into ZrO_2_ lattice and formed t and t′ phases. In addition, the positions of the diffraction peaks shift to high angles with the content of Yb^3+^ increasing (Fig. 1b), which means cell shrinkage. To further investigate the effect of Yb and Y co-doped on phase composition and crystal structure, GSAS software was applied to refine the XRD patterns^28,29^. By comparing the phase content of t phase and t′ phase shown in Fig. 1c, the content of t′ phase increased from 47.5 to 55.5% with the increase of Yb^3+^. The increase of t′ phase is beneficial for improving phase stability of ceramics. Figure 1d displays the tetragonality of t and t′ phase, it can be found that the tetragonality of t and t′ phase showed an opposite trend with the increase of Yb^3+^, and the addition of Yb^3+^ has a greater influence on the tetragonality of t phase. The reduction of tetragonality of t phase is beneficial to inhibit the phase transition from the t phase to the m-phase.

**Figure 2:**
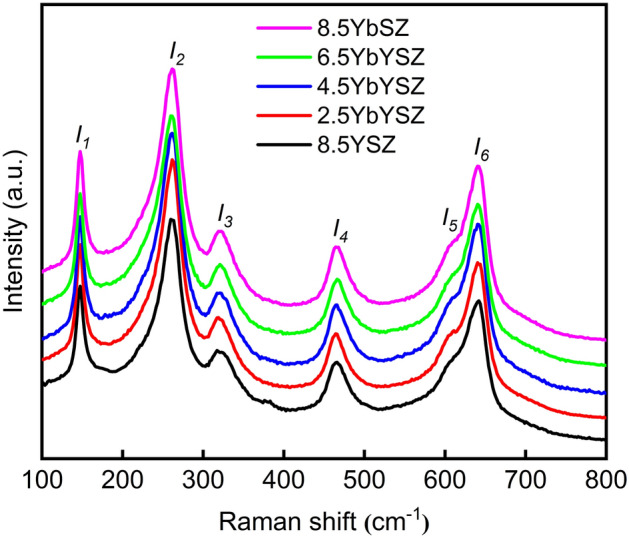
Raman spectra of *x*YbYSZ (*x* = 0, 2.5, 4.5, 6.5, and 8.5) ceramics.

Raman spectra is sensitive to chemical bonds and other short-range ordered structures in the crystal^30^. Therefore, Raman spectra of *x*YbYSZ (*x* = 0, 2.5, 4.5, 6.5, and 8.5) ceramics showed in Fig. 2 is used to analyze the lattice distortion of samples. Raman spectra of all ceramic samples consist of six vibration modes related with tetragonal phase and metastable tetragonal phase, and no monoclinic phase was detected^26,31,32^. Table 1 displays Raman shift of all ceramics. The incorporation of Yb^3+^ has a greater effect on Raman shift of *I*_5_, and the coexistence of Yb^3+^ and Y^3+^ ceramic samples have much lower Raman shift. *I*_5_ is related with chemical bond vibration mode of O–O coupling. Therefore, the *x*YbYSZ (*x* = 2.5, 4.5 and 6.5) ceramics samples are easier to form larger-scale defect clusters, which can improve the resistance of phase transformation controlled by diffusion.

**Table 1:** Raman shift of xYbYSZ (x = 0, 2.5, 4.5, 6.5, and 8.5) ceramic samples.

Samples	Raman shift (cm^−1^)					
	*I*_1_	*I*_2_	*I*_3_	*I*_4_	*I*_5_	*I*_6_
8.5YSZ	147	260	325	467	620	642
2.5YbYSZ	147	260	325	467	618	642
4.5YbYSZ	147	259	324	467	617	642
6.5YbYSZ	147	259	324	465	613	642
8.5YbSZ	147	260	325	467	619	642

### Phase composition of *x*YbYSZ (*x* = 0, 2.5, 4.5, 6.5, and 8.5) after heat treatment for different times

**Figure 3:**
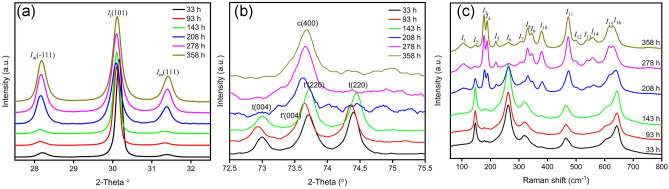
XRD patterns (**a**) 2θ = 27.5°–32.5°, (**b**) 2θ = 72.5°–75.5°, and (**c**) Raman spectra of 8.5YSZ ceramics after heat treatment for different times.

Figure 3 represents the variation of XRD patterns and Raman spectra of 8.5YSZ ceramics after heat-treated with 33, 93, 143, 208, 278 and 358 h. According to Fig. 3a, the intensities of m(− 111) and m(111) peaks increased dramatically after heat treatment for 143–208 h. Furthermore, after heat treatment for 208 h, the characteristic peaks of t and t′ phases of the tetragonal phase were hardly observed (see Fig. 3b). According to the phase diagram of Y_2_O_3_-ZrO_2_^33^, 8.5YSZ was located in the coexisting phase region of the t and c phases. Therefore, the metastable t′ phase is decomposed to the equilibrium t and c phases, and then the t phase transforms to the m phase when the ceramics were heat-treated for a long time. The Raman spectra shown in Fig. 3c further displays that the relative peaks of m phase are appeared with the passage of heat treatment time.

**Figure 4:**
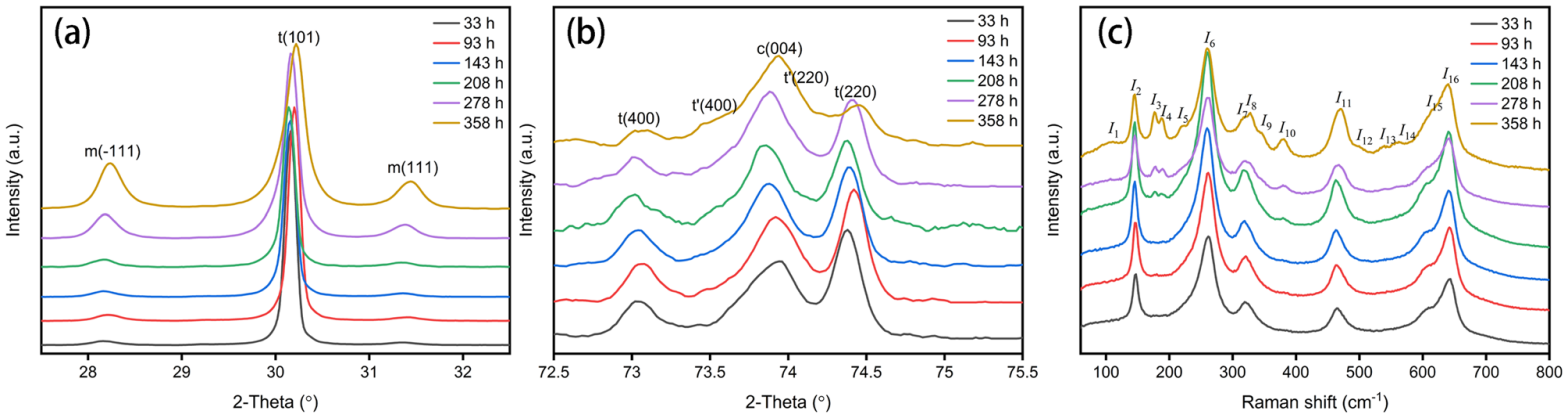
XRD patterns (**a**) 2θ = 27.5°–32.5°, (**b**) 2θ = 72.5°–75.5°, and (**c**) Raman spectra of 6.5YbYSZ ceramics after heat treatment for different times.

According to the previous discussion on the influence of the coexistence of Yb^3+^ and Y^3+^ on the crystal, 6.5YbYSZ ceramic sample is special compared with other ceramic samples. Therefore, Fig. 4 exhibits the variation of XRD patterns and Raman spectra of 6.5YbYSZ ceramic sample after heat-treated different time. It can be seen from Fig. 4 that the changes of XRD patterns and Raman spectra of 6.5YbYSZ ceramic sample are the same as 8.5YSZ. Meanwhile, the characteristic peaks of t and t′ phases shown in Fig. 4 can also be observed after heat treatment for 358 h. Therefore, 6.5YbYSZ ceramic has better phase stability than 8.5YSZ. This result is consisting with above discussion about crystal.

### Variation of monoclinic phase composition of *x*YbYSZ (*x* = 0, 2.5, 4.5, 6.5, and 8.5) after heat treatment for different times

Monoclinic phase is an important factor to estimate the stability of YSZ ceramic materials. XRD is often used to detect the existence of m phase according to Garvie and Nicholson’s equation^34,35^:

1 $${X}_{m}=\frac{{I}_{m}\left(\overline{1 }11\right)+{I}_{m}\left(111\right)}{{I}_{m}\left(\overline{1 }11\right)+{I}_{m}\left(111\right)+{I}_{t}\left(101\right)}$$

where ${I}_{p}^{hkl}$ is the area of the diffraction peak related to the (hkl) crystal plane.

**Figure 5:**
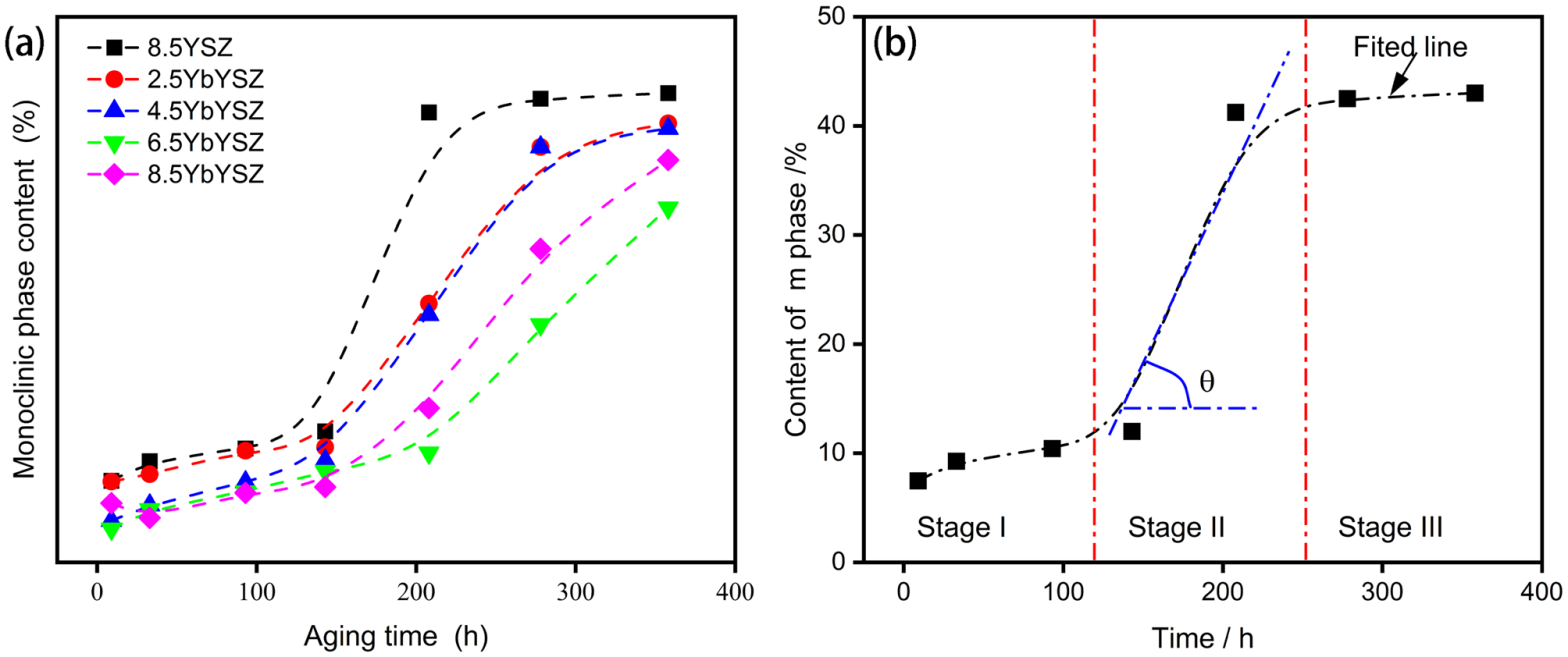
The variation of m phase content of xYbYSZ ceramics (**a**) and fitted line of the m phase content of 8.5YSZ ceramic (**b**) after heat treatment at 1300 °C for 9, 33, 93, 143, 208, 278, and 358 h.

Figure 5 is the variation of monoclinic phase of *x*YbYSZ (*x* = 0, 2.5, 4.5, 6.5, and 8.5) ceramic samples after heat treatment at 1300 °C for 9, 33, 93, 143, 208, 278, and 358 h. It’s obvious seen that the addition of Yb^3+^ is beneficial for improving phase stability and 6.5YbYSZ ceramic sample has the best behavior. What’s more, the relationship between the content of the monoclinic phase and the heat-treated time presents an “S” curve. And the change of monoclinic phase content with different heat-treated time can be divided into three stages (as shown in Fig. 5b): (I) slow increase stage, (II) approximately linear increase stage, and (III) saturation stage. By comparing phase composition of *x*YbYSZ (*x* = 0, 2.5, 4.5, 6.5, and 8.5) ceramic samples after heat treatment for 33, 93, 143, 208, 278, and 358 h shown in Figs. 3 and 4, all ceramic samples have similarity transition process.

Therefore, monoclinic phase variation of 8.5YSZ ceramic sample was discussed to explore the formation reason of “S” curve. Comparing the XRD patterns of 8.5YSZ shown in Fig. 5b, Stage I was processed with two transition processes. One is the transition from t′ phase to t and c phase, and the other one is the transition from initial t phase to m phase. Therefore, Stage I is controlled by the stability of t′ phase and t phase. Therefore, the content of m phase in Stage I gradually increases with the prolongation of heat treatment time. The characteristic peaks of t′ phase vanished and c phase appeared. While in Stage II, the content of m phase sharply increases, and the characteristic peaks of t phase disappear. The characteristic peaks of c phase have little difference in this stage due to t′ phase exhausted in Stage I. Therefore, Stage II is major occurred transition from t phase to m phase, which belongs to martensitic transformation. In Stage III, all transformable phases are exhausted. The content of m phase gets maximized, and Stage III is almost horizontal.

**Figure 6:**
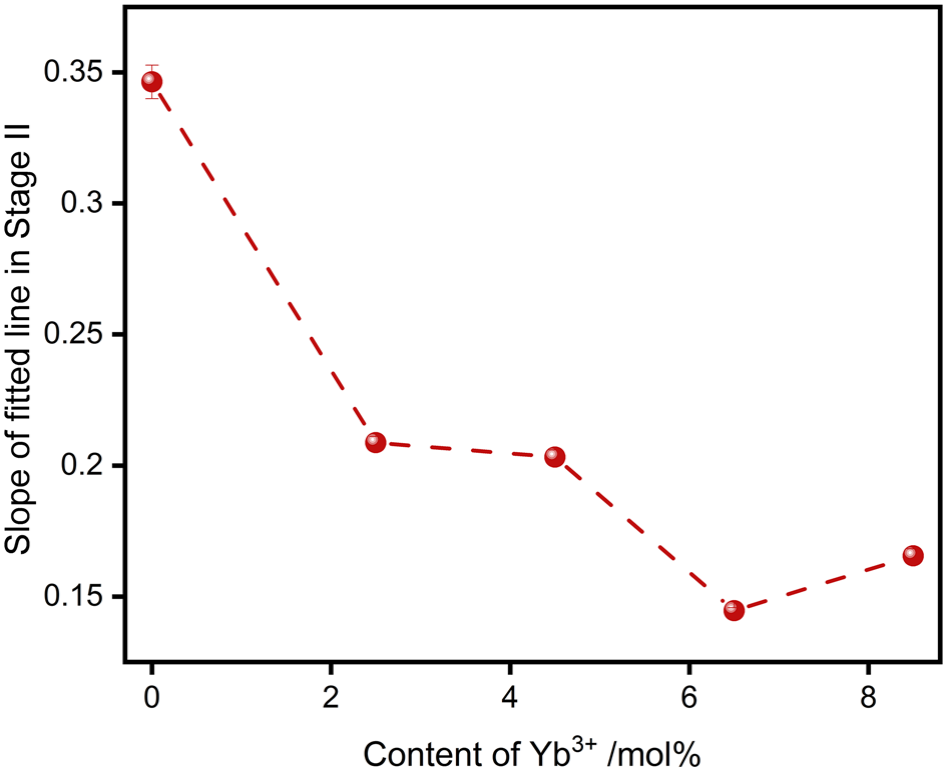
The slope of fitted line in Stage II.

According to previous discussion about crystal structure, the incorporation of Yb^3+^ has effects on O–O coupling, which leads to form large-scale defect clusters difficult to move at high temperature. Meanwhile, the addition of Yb^3+^ is benefit for decreasing the tetragonality of initial t phase, which can improve the stability of t phase. The content of m phase in Stage I of *x*YbYSZ (*x* = 2.5, 4.5, 6.5, and 8.5) ceramic samples is lower than that of 8.5YSZ ceramic sample. And the duration of Stage I of *x*YbYSZ (*x* = 2.5, 4.5, 6.5, and 8.5) ceramic samples is longer than that of 8.5YSZ ceramic sample because of the formation of large-scale defect clusters. In Stage II, the content of the m phase transformed from t phase can be fitted as a line, and the slope of fitted line can reflect indirectly the transformability of t phase. As shown in Fig. 6, the slope of fitted line in Stage II is decrease with Yb^3+^ doped, and 6.5YbYSZ ceramic sample has the lowest slope. The transformable t phase comes from two sources, one is the initial t phase after sintered and the other comes from the transformation of t′ phase. The initial t phase of 6.5YbYSZ ceramic has better stability. Therefore, only the stability of the t-phase derived from the t′-phase transition needs to be discussed. The enhancement of O–O coupling in 6.5YbYSZ ceramics leads to the redistribution of oxygen vacancies in the crystal. Above all, 6.5YbYSZ has the best phase stability after heat treatment at 1300 °C.

## Conclusions

Yb_x_Y_0.085-x_Zr_0.915_O_2-1.5x_ (x = 0, 0.025, 0.045, 0.065, and 0.085) ceramics were prepared using a solid-state reaction method. The content of t′ phase and the tetragonality of t′ phase increase with Yb^3+^ incorporation. What’s more, the addition of Yb^3+^ is good for enhancing O–O coupling, which leads to formation of large-scale defect clusters.

After the *x*YbYSZ (*x* = 0, 2.5, 4.5, 6.5, and 8.5) ceramic samples were heat-treated at 1300 °C for 33, 93, 143, 208, 278 and 358 h, the phase stability of the coexisting Yb^3+^ and Y^3+^ ceramic samples was better, and the m phase change showed an "S"-shaped curve. The “S” curve can be divided into three stages.

The decrease of tetragonality of t phase and O–O coupling was beneficial for improving the phase stability. 6.5YbYSZ ceramic showed the best stability performance after heat treatment at 1300 °C.

## Data Availability

All data generated or analyzed during this study are included in this published article, and the datasets used and analyzed during the current study are available from the corresponding author on reasonable request.
